# Intraluminal Diverticular Duodenal Duplication With Recurrent Abdominal Pain: A Case Report

**DOI:** 10.3389/fped.2022.833304

**Published:** 2022-03-15

**Authors:** Jie Chen, Guo-zuo Xiong, Xiong Tan, Fei Wu, Dong-yang Luo, Qing-qing Zou, Zhihe Deng, Guo-shan Bi

**Affiliations:** ^1^Department of Vascular Surgery, The Second Affiliated Hospital, Hengyang Medical School, University of South China, Hengyang, China; ^2^School of Nursing, University of South China, Hengyang, China

**Keywords:** intraluminal tubular duodenal duplication, diagnosis, subtotal excision, abdominal pain, pediatric surgery

## Abstract

Duodenal duplication is a rare congenital anomaly and may manifest as pancreatitis, gastrointestinal bleeding, abdominal pain, perforation, and obstruction. Here, we present a case of intraluminal diverticular duodenal duplication (IDDD) in a child with recurrent abdominal pain caused by a large hole-like structure in the duodenal bulb. This condition has rarely been reported. An 11-year-old boy presented with recurrent attacks of abdominal pain. Upper endoscopy examination and barium swallowing led to an initial diagnosis of IDDD; this diagnosis was confirmed by operative findings and histopathological signs. He underwent a subtotal excision and duodenal anastomosis. No serious complications occurred following treatment. The patient was followed up for 8 months, and his condition improved without symptoms.

## Introduction

Enteric duplications are uncommon congenital anomalies and account for 5–7% of all gastrointestinal (GI) duplications. By definition, these duplications can be located at any location within the GI tract (1). The terminal ileum is the most common site, whereas the least common site is the duodenum (2). The clinical manifestations of duodenal duplications may be asymptomatic or symptomatic and depend on the stage of development and size. When a duplication is enlarged, bleeding, perforated, or obstructed, patients may experience recurrent episodes of pain, hemorrhage, and pancreatitis (3–5). To diagnose, a duodenal duplication is very challenging because there are no specific characteristics. Although endoscopy and computerized tomography (CT) of barium swallowing are very useful diagnostic tools, the gold standard for diagnosis is still histopathology. With regard to therapy, total removal is still the most optimal form of treatment, although endoscopic surgery and laparoscopy have become popular methods as this technology has advanced (6, 7). Intraluminal diverticular duodenal duplication (IDDD), as a rare form of duodenal duplication, involves an additional diverticulum, which shares part of the wall of the digestive tract with a lesion; this condition has rarely been reported (1, 8). In this study, we report a case of IDDD with a giant pocket-like structure (similar to a “Windsock”) and discuss differential diagnoses and treatments. To our knowledge, this is the first description of such a large IDDD in a child to be reported.

## Case Description

**Figure 1:**
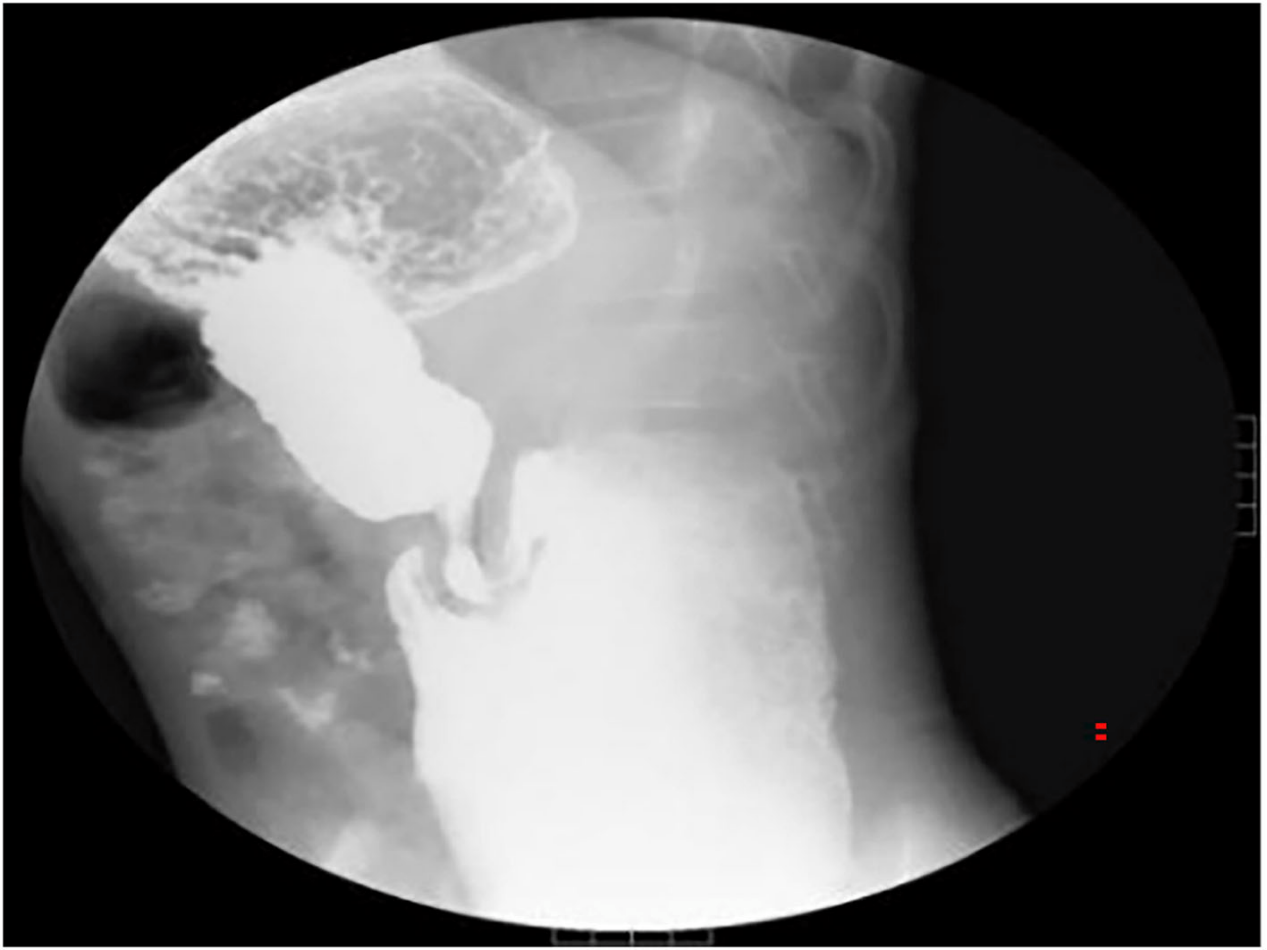
Barium swallowing demonstrated a peculiar defect on the duodenum bulb. The barium-filled structure was huge and had a blind end.

**Figure 2:**
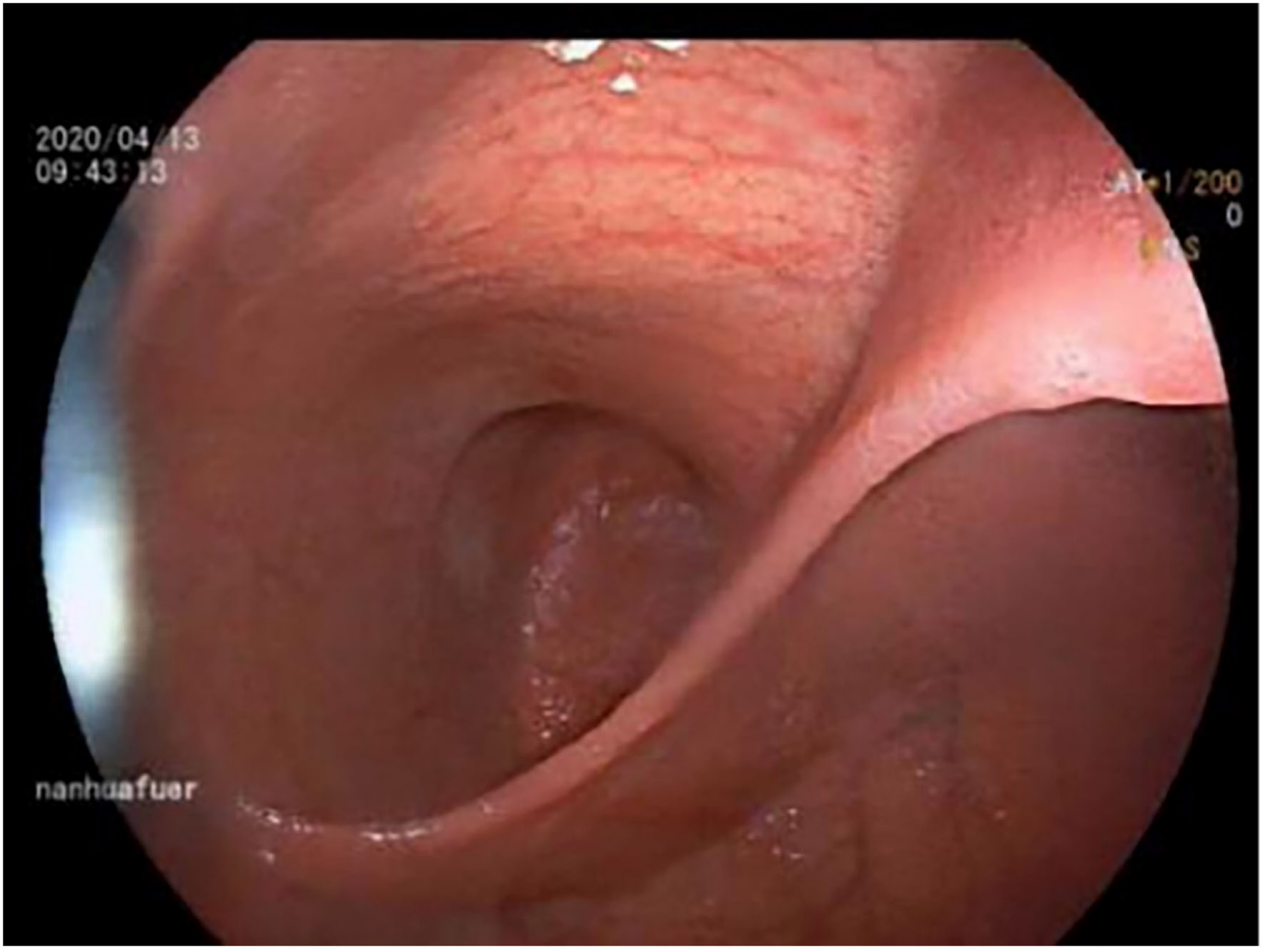
Endoscopy showed a connection with an extra lumen in the duodenal bulb, which shared the common wall with the duodenum bulb.

**Figure 3:**
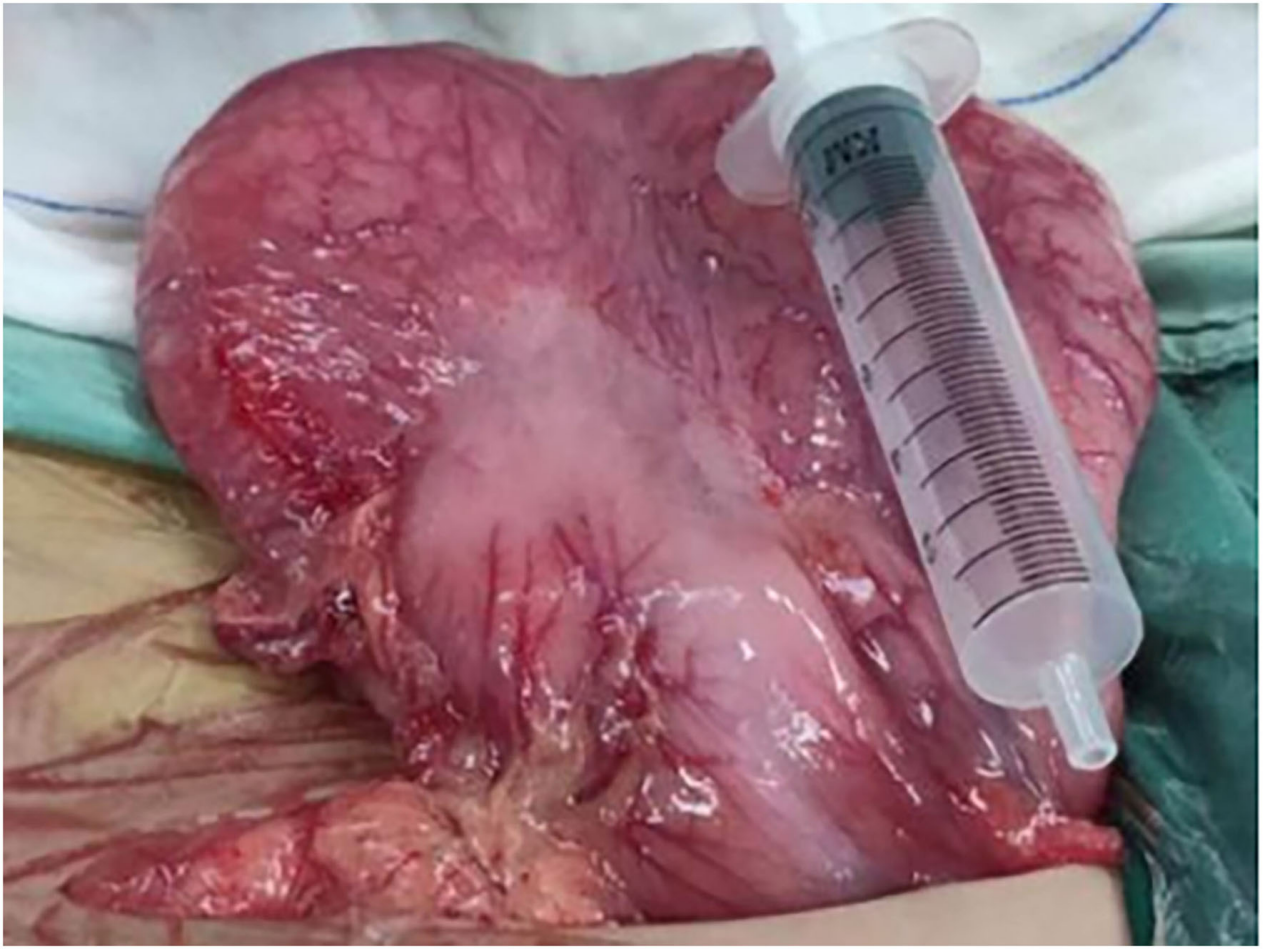
Operative examination revealed a massive lesion that was intimately attached to the big bay of the stomach.

**Figure 4:**
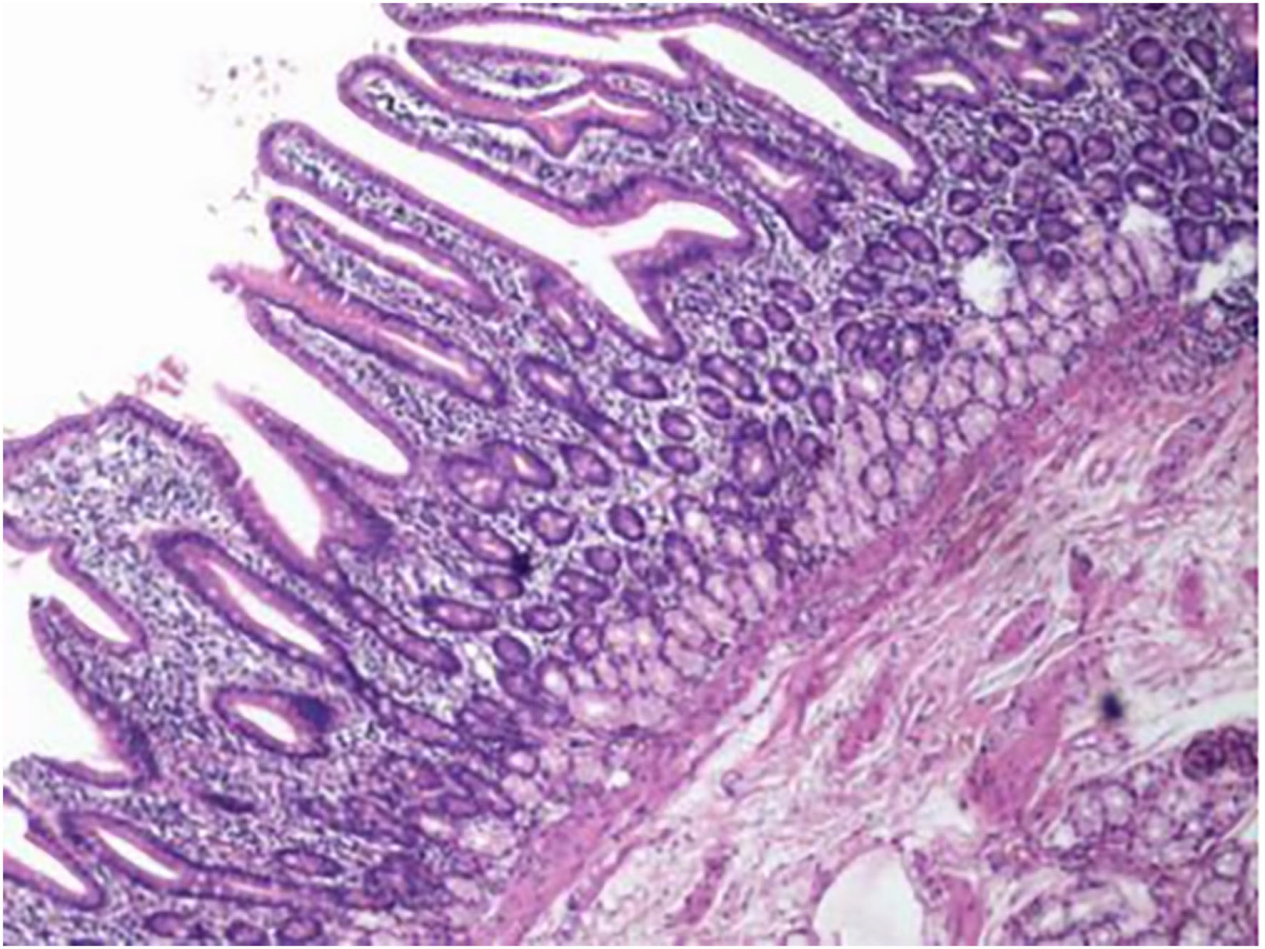
Histological examination of the resected specimen revealed that it featured complete layering with a mucosa, submucosa, muscularis, and serosa.

An 11-year-old boy was referred to our department with a 1-year history of occasional abdominal pain and no vomiting. However, his troublesome pain had recurred and worsened over the last month. The parents told us that their son had experienced poor taste in childhood and denied any history of surgery; there was no family history of this condition. The boy was very thin but his physical examination was unremarkable except for epigastric tenderness. Laboratory examinations also revealed normal levels of carcinoembryonic antigen, bilirubin, and amylase. However, barium swallowing demonstrated that there was a peculiar defect in the duodenum; the barium-filled form was very large and had a blind end (Figure 1). Then, we performed upper GI endoscopy. This showed an additional giant diverticulum-like structure in the duodenal bulb, which shared a common wall with the duodenal bulb (Figure 2), owning a 4 cm width of its opening. The structure was covered with mucous membrane, and the pylorus was abnormally shaped. Fortunately, the major papilla was ~6 cm away from the edge of the diverticulum-like structure. Because of an abundance of food residue, we could not verify the boundary or assess its real size. The diagnostic challenge in this case was whether the disease was a duodenal duplication or duodenal diverticulum; this needed further examination. The abdominal pain was not relieved after admission. On the basis of initial data relating to the duodenal duplication, a total resection was considered the ideal treatment and a management strategy was devised. Therefore, we performed a 6-cm lateral rectus transverse incision and identified a large lesion with a size of 40 × 15 × 10 cm, which was intimately attached to the greater curvature of the stomach; this lesion could not be pulled away from the stomach (Figure 3). The lowest part of the lesion almost reached the bladder. During surgery, we found that the lesion consisted of a true lumen and an extra lumen, which shared part of the wall and formed a membrane-like substance. In addition, the pyloric opening of the stomach was malformed with an irregular pylorus. Under such circumstances, we decided to perform a subtotal ectomy and excised a significant amount of the lesion and the membrane-like substance. Then, we performed pyloroplasty and rebuilt the duodenum. Finally, histological examination confirmed the diagnosis of duodenal duplication: the resected specimen was composed of a complete layering with a mucosa, submucosa, muscularis, and serosa; we referred to this as an IDDD (Figure 4). After surgery, an indwelling gastric tube was placed *via* the nose to the stomach until 4 days after surgery when only a small amount of gastric juice was released. A postoperative abdominal drainage tube showed no obvious fluid exudation, and there was no evidence of celiac disease. The boy drank fluids and progressed to semi-fluids after removal of the tube for 2 weeks. No complications were observed after management and the boy remained asymptomatic at the 8-month follow-up examination except that he ate more and had gained of 2 kg.

## Discussion

Duplications of the tract are congenital lesions and may or may not communicate with the alimentary tract. These duplications can be cystic and tubular in shape although the most common type is the cystic form which accounts for 82% of all GI tract duplications (9). Duodenal duplications are the rarest form of GI malformation (2). The tubular type consists of diverticular and double tracts and can always be identified by the presence of a double tract, an entrance, and an exit; the diverticular type only has an opening. There is another classification method that relies on the grading of intraduodenal cystic lesions as a choledochocele, a duodenal duplication cyst, or an intraluminal duodenal diverticulum (10). Previous studies have described duodenal duplication cysts, which represent 5% of all GI duplications (11, 12). In contrast, intraluminal duodenal diverticulum is rarely reported. A previous review described 96 patients with tract duplication over a 37-year period and found no evidence of tubular duodenal duplications; rather, all duodenal duplications were cystic malformations (11). Furthermore, only a few cases of tubular duodenal duplication or intraluminal duodenal diverticulum have been reported in elderly women (13, 14). Our case was unusual and involved a diverticular and communicating type, which we referred to as an IDDD; this is the first documented instance of a giant IDDD in a child.

The pathogenesis of IDDD remains unclear. It has been suggested that enteric duplications may be caused by the abnormal separation of the embryonic notochord and gastrocoele, a disruption of the recanalization process in the primitive foregut and environmental factors (1). Enteric duplications can be found anywhere in the whole gut and have been found frequently in the distal ileum and rarely in the duodenum. Previous papers reported that duodenal duplications are commonly located in the first or second section of the duodenum (15). Our present case had a lesion in the first segment of the duodenum and had a long history. It is worth noting that enteric duplications are most commonly situated on the mesenteric side of the alimentary tract and share the same blood supply, except for gastric duplications (16). Nevertheless, we found that the IDDD was not located on the mesenteric side of the duodenal bulb and was found near the mesentery and extended to the anterior due to its excessive size.

The diagnosis of these structures is difficult. Although duodenal duplications are congenital malformations that are mainly diagnosed in infants, 33% of cases have been described in adults over 20 years old (1). However, IDDDs usually appear after the age of 30 and vary according to size, type, and location (17). Patients may complain of abdominal pain, nausea, vomiting, or a lack of specific symptoms and signs (18). However, when the lesion obstructs the duodenum or compresses other organs, such as the biliary or pancreatic ducts, the patient may present with recurrent pancreatitis, intestinal obstruction, and pain. Physical examination may reveal no special signs, except for a large cystic form of duodenal duplication. Alternatively, there may be abdominal distention or peritonitis caused by complications. There are no distinguishing differences in terms of laboratory results. For example, levels of amylase and bilirubin may be normal until there is an obstruction in the biliary or pancreatic duct. Because of the long-term stimulation of the diverticulum content and a lack of innervation and peristaltic waves, an IDDD can significantly enlarge and develop a huge lumen (19). The only symptom experienced by our present case was recurrent abdominal pain because of the enormous cavity, mild compression, and communication with the tract, with no obstruction. Therefore, we required delicate instruments to support our diagnosis.

There is a wealth of literature relating to imaging facilities for the diagnosis of IDDD. Contrast-enhanced computed tomography and barium swallowing have been used reliably to detect the site and measure the size of such lesions (20). Endoscopy can accurately assess the location and the intraluminal nature of a duodenal duplication and can even reveal the appearance of an IDDD, which has a double-lumen with an opening into the mouth of the duodenal diverticulum and a mucosal ridge between the two lumina and the opening of the papilla of Vater (21). However, it is very challenging to accurately describe the anatomy using endoscopy alone. There is a need for surgery or laparoscopy to provide more details of the IDDD such as its relationship with surrounding tissues. However, owing to their invasive nature, these techniques are rarely used as a diagnostic method. Notwithstanding, all of these methods target the exterior of an IDDD. The gold standard of diagnosis remains histological examination; using appearance alone, it is not possible to identify an extraluminal duodenal diverticulum from an IDDD. The only difference is that the wall of the duodenal diverticula is characterized by lack of muscular layers, protrudes from the bowel wall, and is not shared by another part of the intestinal tract; IDDDs possess a coat of smooth muscle in their walls and are lined by alimentary tract mucosa (22). Besides diagnosis, histological examination can also detect the origin of the mucous membrane of the intestine. Importantly, several reports have shown that mucous membranes derived from the epithelium of the stomach or pancreas have the potential to become cancerous, although most are benign (23). As observed in our case, the resected specimen was composed of a complete layer of duodenum and operative findings confirmed an IDDD that was adherent to part of the alimentary tract and shared a common wall with the duodenum. Histological findings provided a definite diagnosis, and, fortunately, no heterogeneous epithelium was found.

Some reviews indicate that surgical excision remains the optimal treatment for symptomatic duplications (24). However, conservative therapy remains the best choice if dealing with a very small duplication, if there is no bleeding, if there is no pancreatitis or other symptoms, and if there is a lack of communication with the pancreatic or bile duct. Our present case showed emaciation, and the abdominal pain attacks had become more frequent and severe. Examinations suggested that he had a congenital malformation of the digestive tract. After the patient and his family members were fully informed of his condition, they forcefully requested surgical treatment. However, performing successful surgery in such cases is a significant challenge. Some surgeons favor endoscopic snare excision of the apex of the diverticulum or sac (25). Endoscopic incision of the diverticular wall might be useful in cases where there is a close connection between the attachment of the Vater's papilla, but restenosis has also been associated with this pattern *via* endoscopic incision (6, 25). Several other studies have demonstrated successful surgical resections of the diverticulum *via* laparoscopy as this technique provides a good view, is minimally invasive, causes less damage, and is associated with fast recovery times (11). Noticeably, these experiences are based on adult patients; the role of laparoscopy in the treatment of IDDD in children is not well-defined. Whether surgery can completely or partially remove such lesions depends on the skill of the surgeon and the relationship of the lesion with surrounding organs and tissues. With regard to our present case, complete surgical excision remained the ultimate option. However, laparoscopy resection was not an advisable option. The broad opening and excessive size and mass of the IDDD made the barrel capacity of our case very small. Furthermore, the irregular pylorus and the diverticulum shared walls, thus forming a membrane-like structure. Hence, we selected surgical excision which seemed the best choice for the current condition of the boy. During surgery, we found that the upper side was so intimately attached to the greater curvature of the stomach by sharing common walls that we could not pull these structures apart. We performed a subtotal excision with the upper part remaining and then excised the common walls and performed pyloroplasty. Finally, the operation was successfully completed and fortunately, the patient showed no obvious symptoms or physical signs during the 8-month follow-up period. However, the potential for cancer development and recurrence needs to be considered and lifetime follow-up is required.

In summary, we should consider IDDD as a rare but important differential diagnosis in a child with signs of abdominal pain of unknown origin. Awareness of the endoscopic and radiographic appearance of IDDD, and optimal forms of treatment, is vital points that need to be considered.

## Data Availability Statement

The original contributions presented in the study are included in the article/supplementary material, further inquiries can be directed to the corresponding author.

## Ethics Statement

The studies involving human participants were reviewed and approved by The Second Affiliated Hospital, Hengyang Medical School, University of South China. Written informed consent to participate in this study was provided by the participants' legal guardian/next of kin.

## Author Contributions

G-sB and JC: conception, study design, and draft of the manuscript. JC, G-zX, and XT: surgeons who performed this patient surgery. G-sB, FW, D-yL, Q-qZ, and ZD: references search and revision. G-sB, JC, and XT: manuscript revising. All authors were involved in writing the paper and had final approval of the submitted and published versions.

## Funding

This study was supported by Hunan Province Clinical Medical Technology Innovation Guidance Project (2018SK52202), General Guidance Project of Hunan Provincial Health Commission (20201949), Project of Hunan Provincial Health Commission (B2019111), Hunan Provincial Natural Science Foundation Project (2020JJ5504), Hengyang Science and Technology Bureau Guidance Project (2020161), and Scientific Research Project of Hunan Education Department (19C1620).

## Conflict of Interest

The authors declare that the research was conducted in the absence of any commercial or financial relationships that could be construed as a potential conflict of interest.

## Publisher's Note

All claims expressed in this article are solely those of the authors and do not necessarily represent those of their affiliated organizations, or those of the publisher, the editors and the reviewers. Any product that may be evaluated in this article, or claim that may be made by its manufacturer, is not guaranteed or endorsed by the publisher.

## Abbreviations

DD: duodenal duplications
TDDs: tubular duodenal duplications
IDDD: intraluminal diverticular duodenal duplication
CT: computed tomography.
